# Alternative Forms of Y-Box Binding Protein 1 and *YB-1* mRNA

**DOI:** 10.1371/journal.pone.0104513

**Published:** 2014-08-12

**Authors:** Dmitry N. Lyabin, Alexander N. Doronin, Irina A. Eliseeva, Gelena P. Guens, Ivan V. Kulakovskiy, Lev P. Ovchinnikov

**Affiliations:** 1 Institute of Protein Research, Russian Academy of Sciences, Pushchino, Moscow Region, Russian Federation; 2 Department of Oncology and Radiation Therapy, Moscow State University of Medicine and Dentistry, Moscow, Russian Federation; 3 Engelhardt Institute of Molecular Biology, Russian Academy of Sciences, Moscow, Russian Federation; 4 Department of Computational Systems Biology, Vavilov Institute of General Genetics, Russian Academy of Sciences, Moscow, Russian Federation; University of British Columbia, Canada

## Abstract

The multifunctional eukaryotic protein YB-1 (Y-box binding protein 1) plays a role in DNA reparation, transcription regulation, splicing, and mRNA translation, thereby participating in many crucial events in cells. Its effect is dependent mostly on its amount, and hence, on regulation of its synthesis. Published data on regulation of synthesis of YB-1 mediated by its mRNA 5′ UTR, and specifically on the 5′ UTR length and the presence of TOP-like motifs in this region, are contradictory. Here we report that 5′ UTRs of major forms of human, rabbit, and mouse *YB-1* mRNAs are about 140 nucleotides long and contain no TOP-like motifs mentioned in the literature. Also, we have found that YB-1 specifically interacts with the 5′ UTR of its own mRNA within a region of about 100 nucleotides upstream from the start codon. Apart from YB-1, translation of *YB-1* mRNA in a cell free system gives an additional product with an extended N-terminus and lower electrophoretic mobility. The start codon for synthesis of the additional product is AUC at position –(60–58) of the same open reading frame as that for the major product. Also, in the cell there is an alternative *YB-1* mRNA with exon 1 replaced by a part of intron 1; YB-1 synthesized *in vitro* from this mRNA contains, instead of its N-terminal A/P domain, 10–11 amino acids encoded by intron 1.

## Introduction

The multifunctional nucleocytoplasmic protein YB-1 (Y-box binding protein 1, YB-1, YBX1) is a member of the large family of cold shock domain containing proteins [1]. It is a protein with intrinsically disordered spatial structure that allows its interactions with DNA, RNA, and a large number of proteins. These properties enable YB-1 to play a role in regulation of transcription of many genes, DNA replication and reparation, pre-mRNA splicing in the nucleus, mRNA stabilization, translational control, and mRNA packaging into mRNPs in the cytoplasm [1]. YB-1 is involved in a number of events in the cell, including proliferation, differentiation, and stress response. This makes YB-1 an important factor of embryonic development and underlies its effect on cell adaptation to stress (specifically, acquisition of multiple drug resistance), malignant cell transformation, and metastasis [1], [2].

The wide variety of YB-1 functions dictates the necessity of strict control over its amount in the cell, which depends on the rates of its synthesis and decay. The former is determined by both efficiency of *YB-1* transcription and efficiency of *YB-1* mRNA translation; in turn, the latter depends on both the 3′ UTR [3]–[6] and the 5′ UTR of *YB-1* mRNA [7], [8].

The current study was motivated by the contradictory character of data reported in the literature. First, as reported for human cells KB3-1 and H1299 contain several *YB-1* mRNAs with 5′ UTRs varying from 72 to 331 nucleotides in length [7]. Also, it was shown that YB-1 specifically interacts with the 5′ UTR of its own mRNA, thereby providing negative regulation of its translation. The specific YB-1 binding site was reported to be localized in the initial part (nt 1-200) of the longest, 331 nt *YB-1* mRNA 5′ UTR. However, according to GenBank the length of *YB-1* mRNA 5′ UTRs, though variable, never exceeds 180 nt (Table 1), and for many 5′ UTRs there is no YB-1 binding site in the region indicated in reference [7].

**Table 1:** Comparison of lengths of *YB-1* mRNA 5′ UTRs from different organisms (based on the NCBI database).

Organism	NCBI Reference Sequence	*YB-1* mRNA 5′ UTR length
*Homo sapiens* (human)	NM_004559.3	171 nt
*Oryctolagus cuniculus* (rabbit)	NM_001082785.1	103 nt
*Mus musculus* (mouse)	NM_011732.2	150 nt
*Rattus norvegicus* (rat)	NM_031563.3	124 nt
*Gallus gallus* (chicken)	NM_204414.1	136 nt
*Bos taurus* (bovine)	NM_174815.2	140 nt
*Pan troglodytes* (chimpanzee)	XM_525693.4	182 nt

As we recently showed, the *YB-1* mRNA 5′ UTR is required for mTOR-dependent regulation of YB-1 synthesis in the cell [8]. Importantly, this mode of translation regulation was observed for both the 171 nt human *YB-1* mRNA 5′ UTR with a TOP-like motif [9] and the 103 nt rabbit *YB-1* mRNA 5′ UTR without such a motif [8].

Here, we focused on defining the lengths of 5′ UTRs of *YB-1* mRNAs from different organisms and their impact upon *YB-1* mRNA translation.

## Results

### Defining the length of 5′ UTRs of human, rabbit, and mouse *YB-1* mRNAs

To find the length and nucleotide sequence of 5′ UTRs of endogenous *YB-1* mRNAs, we employed samples of total RNAs from human HeLa and HEK293 cells and rabbit reticulocytes and the technique of rapid amplification of cDNA ends (5′RACE) followed by PCR product cloning into the pJET1.2 vector and sequencing of resulting clones. As seen from Table 2, the 5′ UTR length of human (HeLa and HEK293 cells) and rabbit (reticulocytes) *YB-1* mRNAs ranged from 136 to 139 nucleotides. In one case only (*YB-1* mRNA from HEK293 cells) this value was 118, which could be the result of a reverse transcription interruption at the first stage of RACE, probably caused by CG-rich regions (clusters of C and G nucleotides from 4 to 15 nt each) within the *YB-1* mRNA 5′ UTR.

**Table 2:** Results of 5′RACE experiments.

sample	Sequence	5′UTR length
human HeLa cells	AGTTCGATCGGTAGCGGGAGCGGAGAGCGGACCCCAGAGAGCCCTGAGCAGCCCCACCGCCGCCGCCGGCCTAGTTACCATCACACCCCGGGAGGAGCCGCAGCTGCCGCAGCCGGCCCCAGTCACCATCACCGCAACC	139 nt
human HEK 293 cells_1	AGTTCGATCGGTAGCGGGAGCGGAGAGCGGACCCCAGAGAGCCCTGAGCAGCCCCACCGCCGCCGCCGGCCTAGTTACCATCACACCCCGGGAGGAGCCGCAGCTGCCGCAGCCGGCCCCAGTCACCATCACCGCAACA	139 nt
human HEK 293 cells_2	AGTTCGATCGGTAGCGGGAGCGGAGAGCGGACCCCAGAGAGCCCTGAGCAGCCCCACCGCCGCCGCCGGCCTAGTTACCATCACACCCCGGGAGGAGCCGCGGCTGCCGCAGCCGGCCCCAGTCACCATCACCGCAACC	139 nt
human HEK 293 cells_3	GGAGAGCGGACCCCAGAGAGCCCTGAGCAGCCCCACCGCCGCCGCCGGCCTAGTTACCATCACACCCCGGGAGGAGCCGCAGCTGCCGCAGCCGGCCCCAGTCACCATCACCGCAACC	118 nt
rabbit reticulocytes_1	AGTTCGATCGGTAGCGGGAGCGGAGAGCGGACCCCTGAGAGCCCTGAGCAGCCCCACCGCCGCCGCCGGCCTAGTTACCATCACACCCCGGGAGGAGCCGCAGCTGCCGCAGCCGGCCCCAGTCACCATCACCGCAACC	139 nt
rabbit reticulocytes_2	CGATCGGTAGCGGGAGCGGAGAGCGGACCCCAGAGAACCCTGAGCAGCCCCACCGCCGCCGCCGGCCTAGTTACCATCACACCCCGGGAGGAGCCGCAGCTGCCGCAGCCGGCCCCAGTCACCATTCACCGCAACC	136 nt
rabbit reticulocytes_3	AGTTCGATCGGTAGCGGGAGCGGAGAGCGGACCCCTGAGAGCCCTGAGCAGCCCCACCGCCGCCGCCGGCCTAGTTACCATCACACCCCGGGAGGAGCCGCAGCTGCCGCAGCCGGCCCCAGTCACCATCACCGCAACC	139 nt

**Figure 1:**
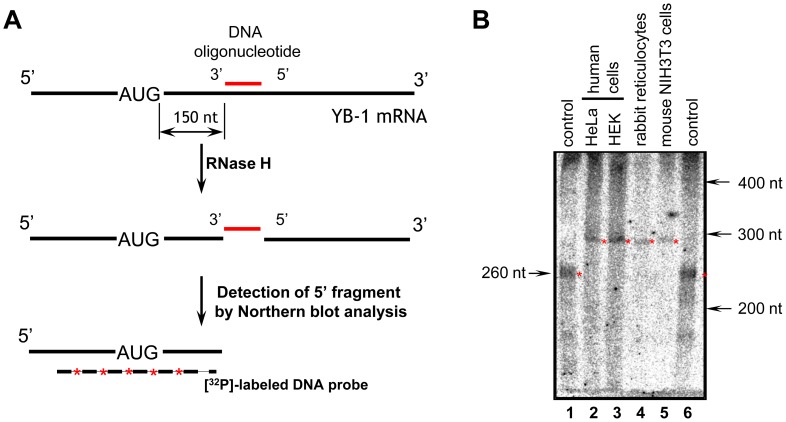
Analysis of the length of 5′UTRs from human, mouse, and rabbit *YB-1* mRNAs. **A**. Scheme of the experiment. Total RNA was annealed with 21 nt DNA oligonucleotide that was complimentary to the *YB-1* mRNA sequence 150–170 nt downstream from the translation start codon, and the sample was treated with RNase H. The reaction products were separated by denaturing polyacrylamide gel electrophoresis, and *YB-1* mRNA 5′-end fragments were detected by Northern blotting with a [^32^P]-labeled DNA probe. **B**. Results of experiment with total RNA of human cells (HeLa and HEK293) – lanes 2 and 3, rabbit cells (reticulocytes) – lane 4, and mouse cells (NIH3T3) – lane 5. As a control (lanes 1 and 6) rabbit *YB-1* mRNA (GenBank, NM_001082785.1) obtained by *in vitro* transcription was used. Radioautograph.

To measure the length of 5′ UTRs by an independent technique, samples of total RNA from human (HeLa and HEK293) and mouse (NIH3T3) cells and from rabbit reticulocytes were treated with RNase H in the presence of a 21 nt DNA oligonucleotide that was complimentary to the *YB-1* mRNA sequence 150–170 nt downstream from the translation start codon (Fig. 1A). The reaction products were separated by denaturing polyacrylamide gel electrophoresis and transferred onto a nylon membrane. Fragments of *YB-1* mRNA with the 5′ UTR and a part of the coding sequence were detected using a radiolabeled DNA probe complementary to the 5′-terminal sequence of *YB-1* mRNA. Figure 1B demonstrates that these fragments from different cell lines had similar lengths of about 290 nt (Fig. 1B, lanes 2–5) and were about 30 nt longer than the 263 nt rabbit *YB-1* mRNA fragment from GenBank, which included a 103 nt 5′ UTR, 149 nt of the coding sequence, and an 11 nt portion of the pBluescript KS vector (Fig. 1B, lanes 1 and 6). It should be noted that since the radioactive signal appears quite weak, it is possible that minor fragments (corresponding to 5′ UTRs of some other lengths) could not be detected with this technique.

Thus, it can be concluded that the length of 5′ UTRs of the major forms of *YB-1* mRNAs from different mammalian cell lines and organisms varies slightly and amounts to about 140 nucleotides.

It is worth noting that the YB-1 transcription start site (TSS) is of a “broad” type spanning dozens of nucleotides. However, the lengths of the 5′ UTRs detected by 5′RACE in this study agree well with the GENCODE annotation [10], with the major transcription signal detected by TSSSeq (presented in dbTSS) [11], and with recent HeliScopeCAGE data from multiple cell types (FANTOM5 project) [12]. Unfortunately, data in existing common databases (such as RefSeq or UCSC) [13] do not agree well with the experimentally verified TSS [14].

### Verification of specificity of the interaction of YB-1 with the *YB-1* mRNA 5′ UTR

**Figure 2:**
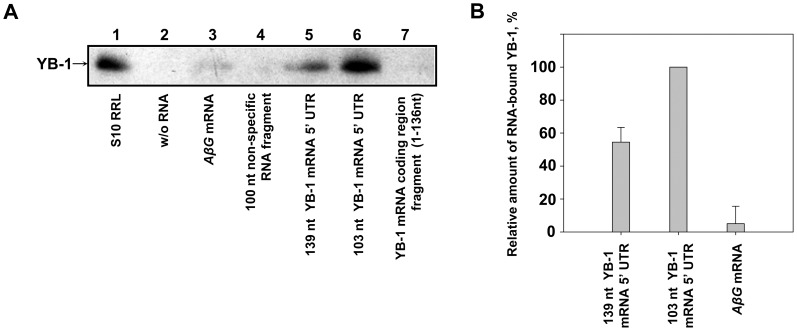
Specificity of YB-1 interactions with *YB-1* mRNA 5′ UTR. **A**. Streptavidin Sepharose-immobilized biotinylated RNA fragments (*AβG* mRNA, 100 nt nonspecific RNA fragment, 139 nt *YB-1* mRNA 5′ UTR, 103 nt *YB-1* mRNA 5′ UTR, and 136 nt *YB-1* mRNA coding region fragment, 15 pmol each, were incubated with 300 µl of rabbit reticulocyte lysates. RNA-bound proteins were eluted, separated by SDS-PAGE, transferred onto a nitrocellulose membrane, and detected with anti-YB-1 antibodies. Lane 1 – rabbit reticulocyte lysate as a marker, lane 2 – control experiment without (w/o) RNA, lane 3 – *AβG* mRNA, lane 4–100 nt nonspecific RNA fragment, lane 5–139 nt *YB-1* mRNA 5′ UTR, lane 6–103 nt *YB-1* mRNA 5′ UTR, lane 7–136 nt *YB-1* mRNA coding region fragment. **B**. YB-1-specific bands from lanes 3, 5, and 6 were quantified using ImageJ software, and RNA-bound YB-1 levels were normalized to the quantity of RNA used (in moles). The amount of 103 nt 5′ UTR-bound YB-1 was taken as 100%. Values are means of three independent experiments. Errors are two standard deviations.

It was reported previously [7] that in human cells YB-1 interacts with a 200 nt 5′-terminal sequence within a 331 nt 5′ UTR of its own mRNA. According to GenBank data and our experimental results reported above, no such sequence can be found in human, rabbit, and mouse *YB-1* mRNAs. Nevertheless, we checked whether or not YB-1 specifically interacted with the 139 nt 5′ UTR of its own mRNA. For this purpose, biotinylated fragments of *YB-1* mRNA containing either truncated (103 nt) or full-length 5′ UTR, as well as control *AβG* RNA(RNA of about 1200 nt in length with actin leader and a GFP fragment), a 100 nt nonspecific RNA fragment, and a 136 nt fragment of the *YB-1* mRNA coding region were immobilized on streptavidin-Sepharose and used to adsorb rabbit reticulocyte lysate proteins. Biotinylated RNA-bound proteins were eluted, separated by SDS-PAGE, and transferred onto a nitrocellulose membrane. YB-1 was detected using antibodies against its C-terminal peptide. As seen in Figure 2A, YB-1 specifically interacted with both full-length and truncated 5′ UTRs of its own mRNA (lanes 5 and 6) but very inefficiently with control RNAs (lanes 3 and 4) or the fragment of *YB-1* mRNA coding region (lane 7). Of note, YB-1 showed a higher affinity for the truncated *YB-1* mRNA 5′ UTR as compared to the full-length one (Fig. 2B), probably because the YB-1 binding site was partially occluded by additional secondary structure when within the larger 5′ UTR fragment. An alternative explanation is that in the case of the more extended 5′ UTR fragment, YB-1 binding was prevented by other proteins specifically interacting with its additional part.

Thus, YB-1 can specifically interact with both full-length and truncated 5′ UTR of its own mRNA within a region of about 100 nucleotides immediately upstream of the start codon.

### Comparison of translational activities of 5′-truncated and full-length *YB-1* mRNAs in a cell-free translation system

**Figure 3:**
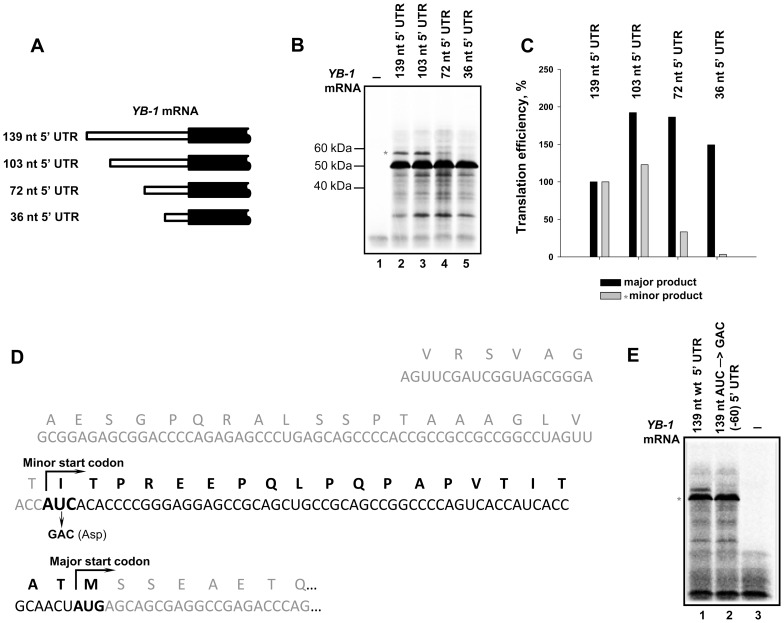
Translation of full-length and 5′-truncated forms of *YB-1* mRNA in the cell-free translation system. **A**. Scheme of *YB-1* mRNAs with 5′UTRs of different length used in the cell-free translation system. **B**. 0.1 pmol of C^+^A^+^ *YB-1* mRNAs with 5′ UTRs of various length (139 nt – lane 2, 103 nt – lane 3, 72 nt – lane 4, 36 nt – lane 5) were translated in the rabbit reticulocyte cell-free system in the presence of [^35^S]-Met. [^35^S]-labeled translation products were resolved by SDS-PAGE and visualized by autoradiography. Lane 1 –translation system without exogenous mRNA. **C**. The relative amount of radioactivity in the bands (**B**) was determined using a Packard Cyclone Storage Phosphor System (Packard Instrument Company, Inc.) The level of translation of 139 5′UTR *YB-1* mRNA was taken to be 100%. **D**. Nucleotide sequence of the 5′-terminal *YB-1* mRNA fragment and its encoded amino acid sequence. An additional amino acid sequence synthesized from AUC at position –(60–58) relative to the major start codon is shown in bold. Mutation in the putative start codon is indicated. **E**. 0.1 pmol of C^+^A^+^ *YB-1* mRNAs with 140 nt WT 5′ UTR (lane 1) or 139 nt AUC→GAC (−60) 5′ UTR (lane 2) were translated in the rabbit reticulocyte cell-free system in the presence of [^35^S]-Met. [^35^S]-labeled translation products were resolved by SDS-PAGE and visualized by autoradiography. Lane 3 shows the translation system without exogenous mRNA.

So, we proved that rabbit, mouse, and human *YB-1* mRNA 5′ UTRs are about 140 nt long. This means that our previous experiments [6], [15] used *YB-1* mRNA with an incomplete 5′ UTR. Hence, we missed the possible effect of this lacking sequence on translation of *YB-1* mRNA. A question arises as to how full-length *YB-1* mRNA is translated in a cell-free system and whether its translational activity differs from that of 5′-truncated *YB-1* mRNA. To answer this question, we generated a construct for synthesizing human *YB-1* mRNA with a 139 nt 5′ UTR. The construct producing rabbit *YB-1* mRNA with a 103 nt 5′ UTR was generated previously [15] (Fig. 3A). Both forms of capped *YB-1* mRNA were used as templates in a cell-free system based on rabbit reticulocyte lysate pretreated with micrococcal nuclease in the presence of [^35^S]-methionine. The [^35^S]-labeled translation products were separated by SDS-PAGE and detected by autoradiography. As seen from Figures 3B and 3C, in the cell-free system translation of truncated *YB-1* mRNA resulted in a slightly larger amount of YB-1 (major product) (Fig. 3B: cf. lanes 2 and 3; Fig. 3C). Additionally detected fragments of higher electrophoretic mobility probably resulted from translation cessation due to partial fragmentation of this mRNA. It is of particular interest that both of the *YB-1* mRNAs gave an additional product of lower electrophoretic mobility (marked with *) that amounted to about 5% of the major product. This additional product could probably be initiated from a noncanonical (minor) codon in the same open reading frame as the major product. In an experiment on *YB-1* mRNAs with truncated (72 nt and 36 nt)5′ UTRs (Fig. 3A), we found that synthesis of the additional product notably decreased with shorter 5′UTR lengths (down to 72 nucleotides), and it ceased completely with a 5′ UTR as short as 36 nucleotides (Fig. 3B: lanes 4 and 5; Fig. 3C). Supposedly, the start codon for synthesis of the additional product could be positioned between nucleotides 36 and 72 within the 5′ UTR, and specifically, it could be AUC at the position –(60–58) within the same open reading frame as the major product. To test this assumption, we generated mutant *YB-1* mRNA with GAC substituted for the AUC codon (Fig. 3D). The mutation resulted in complete disappearance of the additional product (Fig. 3E) without affecting synthesis of the major product. According to calculations, the additional product must be larger by 20 amino acid residues, i.e., by about 2 kDa. However, as follows from the electrophoregram, the shift (relative to the major product) was ∼7 kDa. This difference might be explained by anomalous electrophoretic mobility of extended YB-1, which is known to be typical of the major YB-1 form too (+15 kDa).

Thus, synthesis of YB-1 as the major product of *YB-1* mRNA translation can be accompanied by an additional form of YB-1 with an extended N-terminus (Fig. 3D). It should be noted that in some cell lysates, highly specific antibodies against the YB-1 C-terminal peptide revealed a protein with electrophoretic mobility similar to this additional YB-1 (Fig. S1).

### Alternative forms of *YB-1* mRNA

**Figure 4:**
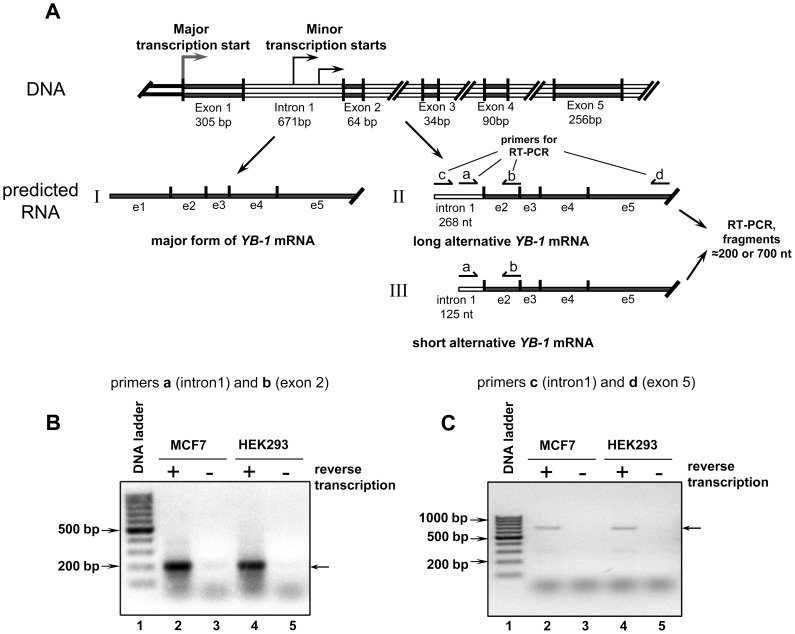
Search for alternative forms of *YB-1* mRNA in the cell. **A**. Scheme of predicted alternative *YB-1* mRNA. **B and C**. Total RNAs from MCF7 (lanes 2 and 3) and HEK293 (lanes 4 and 5) were used in the reverse transcription reaction followed by PCR (lanes 2 and 4) or in PCR only (lanes 3 and 5) with primers **a** and **b** specific to intron 1 and exon 2 of the *YB-1* gene (**B**) or primers **c** and **d** specific to intron 1 and exon 5 of the *YB-1* gene (**C**). PCR products were resolved by electrophoresis in 2% agarose gel stained with ethidium bromide. Lane 1 shows the DNA ladder.

According to a full genome analysis of transcription start sites [16], the *YB-1* gene has alternative TSSs, two of which were found in the first intron of *YB-1* at positions 404 and 547 (Fig. 4A). We verified the existence of mRNAs synthesized from these TSSs using total RNA isolated from human HEK293 and MCF7 cells. The samples were treated with DNase I and subjected to reverse transcription with primer **d** complimentary to exon 5 of *YB-1* mRNA followed by PCR with primer **b** and primer **a** complimentary to intron 1 (nt 547–570 within intron 1) (see Fig. 4A). This revealed both truncated and full-length forms of alternative *YB-1* mRNA. As a control PCR template, total RNA without reverse transcription was used. As seen from Figure 4B, PCR products corresponding to mRNA synthesized from the TSS of *YB-1* intron 1 were detected in total RNA from HEK293 and MCF7 cells (lanes 2 and 4, respectively), but not in controls (lanes 3 and 5, respectively). As expected, the PCR products were about 200 bp in size, and their sequencing showed that the alternative minor *YB-1* mRNA contained a part of intron 1 followed by exon 2 (Fig. S2). To rule out the possibility of RT-PCR product synthesis from the primary transcript, our next experiment used primer **c** complimentary to intron 1 (nt 404–423 within intron 1) and primer **d** complimentary to exon 5 (Fig. 4A), which predictably gave a PCR product of about 700 bp in size (Fig. 4C, lane 1 and 3). This means that this cDNA product was synthesized from spliced mRNA beginning with intron 1.

**Figure 5:**
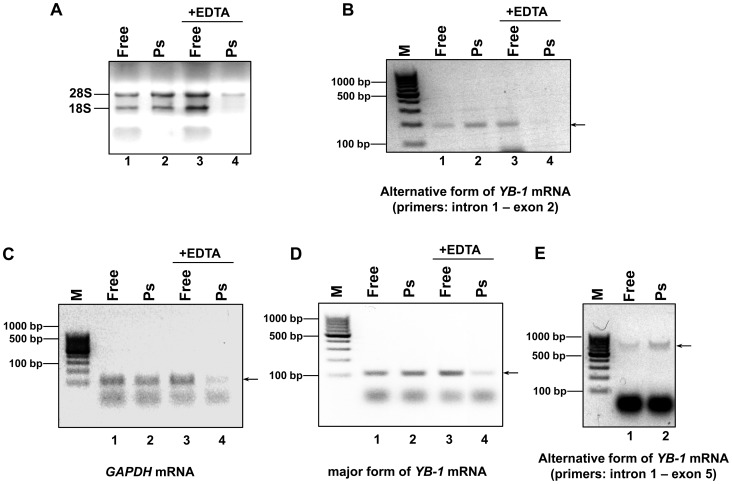
Search for alternative forms of *YB-1* mRNA in the polysome fraction. MCF7 cell lysate with or without EDTA was spun through a 50% sucrose cushion at 90,000 rpm in a TLA-100 centrifuge (Beckman) for 13 min to separate postpolysomal supernatant from polysomes. Total RNA from postpolysomal supernatant and polysome fractions (resuspended pellets) was extracted with TRIzol and LiCl reprecipitated. A portion of total RNA was taken for subsequent analysis. The rest was used for reverse transcription and PCR with gene-specific primers. The total RNA samples and DNA PCR products were subjected to agarose gel electrophoresis and stained with ethidium bromide. **A**. Total RNA (4 samples). **B-E**. DNA PCR products from: alternative form of *YB-1* mRNA (primers **a** and **b** for PCR) (**B**); *GAPDH* mRNA (**C**); major form of *YB-1* mRNA (**D**); alternative form of *YB-1* mRNA (primers **c** and **d** for PCR) (**E**). Arrows indicate the expected PCR products.

Having proved the existence of alternative *YB-1* mRNA(s), we proceeded to verify its translation in the cell. This could be evidenced by the presence of an alternative form of *YB-1* mRNA in polysomes. MCF7 cell lysate was centrifuged through 50% sucrose to give free mRNPs mostly in the supernatant and polysomal mRNPs in the pellet. Isolation of the total RNA from these fractions was followed by detection of an alternative form of *YB-1* mRNA (or *GAPDH* mRNA and major *YB-1* mRNA as controls) by RT-PCR with transcript-specific primers (see Materials and Methods) (Fig. 5). Figure 5A demonstrates the efficiency of lysate fractionation into polysome and postpolysomal fractions. In the absence of EDTA, the majority of the 28S rRNA and 18S rRNA was observed in the polysome fraction. Addition of 30 mM EDTA to the cell lysate resulted in ribosome dissociation and transition of almost the entire rRNA into the postpolysomal fraction (free mRNP), while only a small part of the 28S rRNA was detected in the polysome fraction.

As seen from Figure 5, the polysome fraction contained an alternative form of *YB-1*

mRNA and the major form of *YB-1* mRNA and *GAPDH* mRNA as controls (Fig. 5B, 5C, and 5D, lane 2). Addition of EDTA to the cell lysate resulted in transition of almost the entire mRNA into the free mRNP region (cf. lanes 3 and 4 in Figs. 5B, 5C, and 5D, respectively). The presence of an alternative form of *YB-1* mRNA in polysomes was also demonstrated in similar experiments using a reverse primer complimentary to exon 5 (Fig. 5E). Together, these results are evidence for the presence of *YB-1* mRNA in polysomes, which, in turn, indicates its probable translation in the cell.

**Figure 6:**
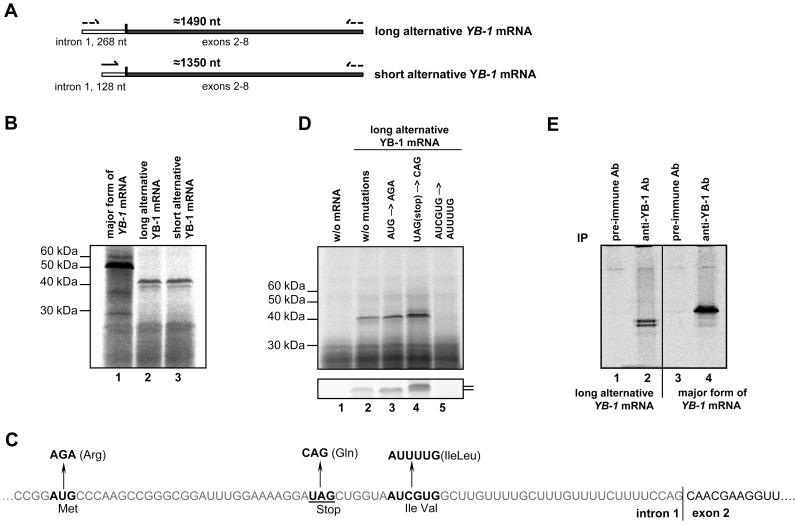
Analysis of translation of alternative *YB-1* mRNAs. **A**. Scheme of long and short alternative *YB-1* mRNAs. **B**. 0.1 pmol of C^+^A^+^ *YB-1* mRNAs with 140 nt WT 5′ UTR (lane 1) or long alternative *YB-1* mRNA (lane 2) or short alternative *YB-1* mRNA (lane 3) were translated in the rabbit reticulocyte cell-free system in the presence of [^35^S]-Met. [^35^S]-labeled translation products were resolved by SDS-PAGE and visualized by autoradiography. **C**. Scheme of mutations in the putative start and stop codons in intron 1 of alternative *YB-1* mRNA. **D**. 0.1 pmol of C^+^A^+^ long alternative *YB-1* mRNA (lane 2) or long alternative *YB-1* mRNA with AUG→AGA mutation (lane 3), or UAG(stop) →CAG mutation (lane 4), or AUCGUG→AUUUUG mutation (lane 5) was translated in the rabbit reticulocyte cell-free system in the presence of [^35^S]-Met. [^35^S]-labeled translation products were resolved by SDS-PAGE and visualized by autoradiography. Lane 1 shows the translation system without exogenous mRNA. The lower panel shows the same samples after longer electrophoresis. **E**. Translation reaction mixture with long alternative *YB-1* mRNA or WT *YB-1* mRNA was used for immunoprecipitation with preimmune antibody or YB-1 antibody. Proteins bound to antibodies were resolved by acid–urea PAGE, and [^35^S]-labeled proteins were detected by autoradiography.

Next, we generated plasmid constructs for synthesis of alternative *YB-1* mRNAs *in vitro* (Fig. 6A) and checked whether these mRNAs could serve as templates for protein synthesis in the cell-free translation system. Strictly speaking, existence of a short form of *YB-1* mRNA in the cell cannot be recognized as proved, because its cDNA could have been synthesized from an alternative full-length *YB-1* mRNA. Nevertheless, it was shown that both full-length and truncated *YB-1* mRNAs could be translated in the cell-free system to give a protein with an electrophoretic mobility of about 40 kDa, which is smaller than the estimated molecular weight of YB-1 (Fig. 6B). This means that translation of the both alternative *YB-1* mRNAs was initiated from the same start codon, probably located between nt 547 and 671 within intron 1, although generally codons of this region are unfit for translation initiation. Specifically, the best suited codon AUG is in an unfavorable context, and besides, there is a stop codon in the same frame (Fig. 6C). However, as shown by toe printing (Fig. S3), the 48S complex can be formed at this codon, and hence it cannot be ruled out that protein synthesis starts there and proceeds uninterrupted through the stop codon. But it seems more likely that synthesis of a product as large as 40 kDa starts at one of two adjacent noncanonical codons (AUC and/or GUG) located in the 3′-terminal part of intron 1. They are in the optimal context and within the same open reading frame as the sequence in exon 2 (Fig. 6C). To identify the start codon for translation of the alternative *YB-1* mRNA, we constructed *YB-1* mRNA mutants where AUG was replaced by AGA (Arg), or glutamic CAG was substituted for UAG (termination codon), or AUUUUG took the place of AUCGUG (the most probable translation start codons) (Fig. 6C). These mutants, as well as unmodified alternative *YB-1* mRNA (control), were translated in the rabbit reticulocyte cell-free system in the presence of [^35^S]-methionine. The [^35^S]-labeled products were separated by SDS gel electrophoresis and detected by autoradiography. As seen from Fig. 6D, neither replacement of AUG nor UAG had a negative effect on synthesis of the alternative YB-1 (cf. lanes 2, 3, and 4). Replacement of the two adjacent codons, which probably were start codons, resulted in complete cessation of synthesis of this protein (lane 5). It is worth noting that CAG substitution for UAG resulted in synthesis of an additional product with slightly lower mobility and higher intensity (Fig. 6D, lower panel, lane 4). This supports the assumption that translation initiation begins at the AUG codon to give a 1 kDa larger protein. Besides, replacing AUG with AGA resulted in a higher level of protein synthesis (lane 3). Presumably this mutation enhanced the probability of usage of the start codon within the major open reading frame. On the whole, translation of an alternative *YB-1* mRNA can be described as follows. The scanning 40 S subunit reaches the AUG codon and initiates translation that stops at the nearest stop-codon UAG having produced a 10 a.a. long peptide. Then, the ribosome (or its 40 S subunit after 60 S dissociation) most probably finds another closely positioned start codon (AUC or GUG) and initiates synthesis of the alternative YB-1.

Thus, it can be concluded that a region within intron 1 initiates translation to produce a 10-11 amino acid peptide that precedes the YB-1 sequence coded by *YB-1* mRNA beginning with exon 2; this YB-1 sequence includes the cold shock domain and the C-terminal domain typical of the major YB-1. To detect the YB-1 C-terminus in the resultant product of alternative *YB-1* mRNA translation, we used immunoprecipitation of proteins synthesized in the cell-free system with major and alternative *YB-1* mRNAs in the presence of [^35^S]-methionine. The experiment using antibodies against the YB-1 C-terminal peptide detected both the major YB-1 (Fig. 6E, lane 4) and the product of alternative *YB-1* mRNA translation (Fig. 6E, lane 2) in the immunoprecipitate, while that with control preimmune antibodies revealed no [^35^S]-labeled proteins (Fig. 6E, lanes 1 and 3). For alternative *YB-1* mRNA, the translation product was represented by two distinct bands, which probably resulted from the use of acid–urea electrophoresis, because no such doublet was observed in the case of SDS gel electrophoresis.

These results identified a protein which is synthesized from alternative *YB-1* mRNA as YB-1 where the N-terminal A/P domain (55 a.a. long) has been replaced by a 10–11 a.a. sequence coded by intron 1.

## Discussion

Here we report that the length of 5′ UTRs from human, rabbit, and mouse *YB-1* mRNAs is about 140 nucleotides. For some 5′ UTRs the GenBank database gives different values, e.g., for that of rabbit *YB-1* mRNA (NM_001082785.1) or human *YB-1* mRNA (NM_004559.3). Presumably, these are minor forms resulting from a rather broad range of *YB-1* transcription initiation [17]. About 70% of eukaryotic promoters are known to be classified as “broad” ones because their transcription start sites (TSS) range over dozens of nucleotides. There is a probability that the *YB-1* cDNA (mRNA) yielded by rabbit cDNA library cloning in 1995 [18] was truncated, which is supported by the fact that in one of our samples from HEK293 cells 5′RACE revealed a *YB-1* mRNA 5′ UTR of 118 nucleotides in length. We failed to find a 331 nt *YB-1* mRNA 5′ UTR mentioned by Fukuda et al. [7], which either puts in doubt its existence or suggests it for certain cell types only (KB3-1 and H1299). Besides, the major 139 nt 5′UTR does not include a TOP-like motif that was reported by Hsieh et al. [9] as found at the beginning of a 171 nt 5′ UTR of *YB-1* mRNA. Thus, the signal responsible for sensitivity of *YB-1* mRNA translation to inhibitors of the mTOR signaling cascade must be localized elsewhere; according to our finding [8], it belongs to a 100 nt sequence adjoining the start codon within the 5′ UTR.

Our current experiments on specific binding of YB-1 to *YB-1* mRNA 5′ UTR fragments indicate that it occurs within a sequence of about 100 nt preceding the start codon. The functional importance of this event is still to be understood; here we have two hypotheses to propose.

As known, overexpression of YB-1 results in inhibition of translation of some mRNAs, including TOP mRNAs whose translation depends on the mTOR signaling cascade [19]. This suggests a role of YB-1 in translation regulation through the mTOR signaling pathway and the necessity of YB-1 binding to the 5′ UTR of *YB-1* mRNA and to 5′ UTRs of other similar mRNAs to contribute to regulation of their translation.

The other hypothesis is based on the fact that the same YB-1 molecule binds both to the *YB-1* mRNA 5′ UTR and to the regulatory element within the *YB-1* mRNA 3′ UTR [4], [6] and participates in selective inhibition of *YB-1* mRNA translation. This hypothesis, if supported, could explain how YB-1, positioned at the 3′ end, inhibits translation initiation at the 5′ UTR.

We have found that the cell-free translation system with full-length or truncated *YB-1* mRNA yields an additional translation product using as a start codon AUC at the position –(60–58) with respect to the major start codon. It is worth noting that AUC as an alternative start codon has been detected in human and mouse cell lines by ribosome profiling [20], [21]. The possibility of *in vivo* synthesis of extended YB-1 products was reported in the literature. According to David et al. [22], in mouse cardiomyocytes 50 kDa YB-1 was accompanied by 60 kDa YB-1; the amount of the latter gradually increased during postnatal development of the heart ventricle, and in a mature mouse its level exceeded that of the 50 kDa YB-1. As shown, the changing ratio of these two isoforms is functionally important for regulation of expression of the *smooth muscle α-actin(SMaA)* gene.

Besides, in some cell lysates highly specific antibodies against the YB-1 C-terminal peptide revealed a protein with electrophoretic mobility similar to that of the additional YB-1 isoform reported here (Fig. S1). It cannot be ruled out that extended YB-1 isoforms are synthesized from other start codons within the *YB-1* mRNA 5′ UTR, because the whole 5′ UTR can be read in the frame of the major start codon (Fig. 3D).

Lastly, in HEK293 and MCF7 cells we have detected an alternative *YB-1* mRNA that has a part of intron 1 in the position of exon 1. It has been detected in polysomes, which points to the probability of its translation *in vivo*. Interestingly, this mRNA should have a 5′UTR coding for intron1 but not exon 1, and it can serve as a template for protein synthesis in the cell-free system using one of two adjacent triplets AUC and/or GUG as the start codon. In this case the minor RNA can produce N-truncated YB-1 with a 10–11 a.a. sequence (encoded by intron 1) instead of its A/P domain.

Recent high-throughput analysis of cell-specific transcriptional activity performed by the FANTOM Consortium [12] has shown that the intronic YB-1 TSSs are highly active in some cell types including both cancer and normal cells (Fig. S4). Also, the isoform transcribed from the intronic TSS has been included in curated GENCODE annotation [10]. Presumably, there are mechanisms regulating the amount of alternative YB-1 in the cell. This is still to be verified.

## Materials and Methods

### Cell cultures

NIH3T3, HEK293, and MCF7 cells (originally obtained from ATCC) were kindly provided by Dr. Elena Nadezhdina (Institute of Protein Research, Russian Academy of Sciences) and were cultivated in Dulbecco's Modified Eagle's Medium (DMEM) supplemented with 10% fetal calf serum, 2 mM glutamine, 100 U/ml penicillin, and 100 µg streptomycin (PanEco). HeLa cells were cultivated in DMEM/F12 supplemented with 10% fetal calf serum, 2 mM glutamine, 100 U/ml penicillin, and 100 µg streptomycin (PanEco). The cells were incubated at 37°C in a humidified atmosphere containing 5% CO_2_ and passaged by standard methods.

Rabbit reticulocyte samples were obtained from the Institute of Theoretical and Experimental Biophysics, Russian Academy of Sciences, where before use in experiment the rabbits were kept under standard conditions of the barrier zone in compliance with the Program of Care and Use of Animals, and all manipulations with them were performed in compliance with ethical standards in animal research approved by the Institute Commission on Biological Safety and Ethics established on October 3, 2011 (# 173/k).

### Total RNA Isolation and cDNA preparation

Total RNA from cells was extracted by the TRIzol method and treated with DNase I. Reaction mixture (20 µl) contained 1 µg of total HeLa, HEK293, or MCF7 RNA, 100 pmol of oligo(dT)_20_ primer or gene specific primer, 10 u/µl of reverse transcriptase MMuLV RNase H Minus (Fermentas, Lithuania), and appropriate buffer. It was incubated at 42°C for 1 h, phenol extracted, and ethanol precipitated.

### 5′RACE (5′ Rapid Amplification of cDNA Ends)

5′RACE analysis was performed using a Mint RACE cDNA amplification set (Evrogen, Russia) according to the manufacturer's recommendations. Gene specific primers for 5′RACE were 5′-AGGCGCCGCCGATGTGAGG-3′ and 5′- CTGCCCATGGTTGCGGTGATGGTGACTG-3′.

### Detection of mRNA in total RNA and polysomes

An alternative form of *YB-1* mRNA in total RNA was detected using PCR with HEK293 and MCF7 total cDNA (or HEK293 and MCF7 total RNA in a control experiment) as template and primers **a**–5′-TGGAGAGAAAGGGCTGTCAGGTG-3′ complementary to nt 547-570 (within intron 1) and **b**–5′-GATGAAACCATATCCGTTCCTTACATTG-3′ complementary to exon 2, or primers **c**–5′-TCCCTCACGTGCTCTCCGTC-3′ complementary to nt 404-423 (within intron 1) and **d**–5′-CCCATAGGGTCTCCGCATG-3′ complementary to exon 5. PCR products were analyzed by agarose gel electrophoresis. The major form of *YB-1* mRNA, an alternative form of *YB-1* mRNA, and *GAPDH* mRNA in polysomes were detected using PCR with cDNA obtained from polysomal and free mRNPs of MCF7 cells as template and the following primers: for alternative *YB-1* cDNA – the same primer as for the total RNA; for GAPDH cDNA – 5′-ACAGCCTCAAGATCATCAGCAAT-3′ and 5′-ATGGACTGTGGTCATGAGT CCTT-3′; for the major form of *YB-1* cDNA – 5′-ATCACCGCAACCATGGGCAGCGA-3′ complementary to exon 1 and 5′- AGGCGCCGCCGATGTGAGG-3′ complementary to exon 1. PCR products were analyzed by agarose gel electrophoresis.

### Analysis of length of *YB-1* mRNA 5′ UTR

A mixture (10 µl) containing 20 pmol of oligonucleotide (5′-GTTGCGATGACCTTCTTGTCC-3′) complementary to the *YB-1*mRNA sequence at 150 nucleotides from the start codon and 30 µg of total RNA from HeLa, HEK293, or NIH3T3 cells or rabbit reticulocyte was incubated for 5 min at 70°C and cooled to room temperature. Then 2.5 µl of 5× RNase H buffer (200 mM Hepes-KOH, pH 7.6, 300 mM KCl, 50 mM MgCl_2_, 5 mM DTT) and 1 unit of RNase H (Fermentas) were added. The reaction mixture was incubated for 30 min at 37°C, and RNA was analyzed by Northern blotting.

### Northern blot analysis

Total RNA from cells or tissues was separated by electrophoresis on a denaturing 5% polyacrylamide gel. RNA was transferred onto a nylon membrane (Hybond-N, GE Healthcare) and crosslinked using a transilluminator-cross-linker (Vilber-Lourmat) at 0.15 J/cm^2^. Membrane-bound RNA was hybridized to a 240 nt 5′ fragment of *YB-1* cDNA probe (nt 1-240, GenBank U16821.1) labeled with [^32^P]dATP (40 mCi/ml, 2000 Ci/mM; IBCh, Russia) using a DecaLabel DNA labeling kit (Fermentas) in hybridization buffer (0.5 M KH_2_PO_4_/K_2_HPO_4_,pH 7.4, 7% SDS, 10 mM EDTA) at 65°C for 12–16 h. The membrane was washed twice with 2x SSC, 0.1% SDS for 5 min at room temperature (RT), twice with 0.2x SSC, 0.1% SDS for 5 min at RT, twice with 0.2x SSC, 0.1% SDS for 15 min at 42 °C, and twice with 0.1x SSC, 0.1% SDS for 15 min at 68 °C, and analyzed by autoradiography using a Packard Cyclone Storage Phosphor System (Packard Instrument Company, Inc.).

### Plasmid construction and templates for *in vitro* transcription

The pBluescript II SK *YB-1* WT construct containing rabbit *YB-1* cDNA was described earlier [15].

The template for full-length (140 nt 5′ UTR) *YB-1* mRNA *in vitro* synthesis was obtained by PCR with total HeLa cDNA as template. The forward primer was 5′-**TAATACGACTCACT ATAGGG**AGTTCGATCGGTAGCGGGAGCG-3′ and contained T7 promoter sequence (shown in bold). The reverse primer was 5′-CCAAGCTTATTTAAGACCTTTATTAACAGG-3′.

The templates for truncated (103 nt 5′ UTR) *YB-1* mRNA, truncated (72 nt 5′ UTR) *YB-1* mRNA, and truncated (36 nt 5′ UTR) *YB-1* mRNA *in vitro* synthesis were obtained by PCR with total HeLa cDNA as template. The forward primers were 5′-**TAATACGACTCACTATAGGG** CCCCAGAGAGCCCTGA-3′,5′-**TAATACGACTCACTATAGGG**CCTAGTTACCATCACACCCCGGG-3′, and 5′-**TAATACGACTCA CTATAGGG**CAGCTGCCGCAGCCGGCC-3′, respectively. The reverse primer was 5′-CCAAGCTTATTTAAGACCTTTATTAACAGG-3′.

The plasmid pJET 1.2-5′-UTR YB-1_truncated was obtained by ligation of pJET 1.2 vector (Fermentas) with PCR product amplified using the total HeLa cDNA as template and primers 5′-GACTCGAGAGCCCTGAGCAGCCCCAC-3′ and 5′- CTGCCCATGGTTGCGGTGATGG TGACTG-3′.

The plasmid pJET 1.2-5′-UTR YB-1_full was obtained by ligation of pJET 1.2 vector (Fermentas) with PCR product amplified using the total HeLa cDNA as template and primers 5′-GTCTCGAGGGCTTATCCCGCCTGTC-3′ and 5′-CTGCCCATGGTTGCGGTGATGGTGACTG-3′.

The plasmid pJET 1.2 YB-1_coding region was obtained by ligation of pJET 1.2 vector (Fermentas) with PCR product amplified using the pBluescript II SK *YB-1* WT construct as template and primers 5′-ATGAGCAGCGAGGCCGAG-3′ and 5′-TTACTCAGCCCCGCCCTG-3′

The template for AUC→GAC –(60–58) *YB-1* mRNA *in vitro* synthesis was obtained using site-specific mutagenesis by overlap extension. Two PCR products were amplified using the plasmid pBluescript II SK *YB-1* WT as template and primers 1 and 2 (5′-**TAATACGACTCACTATAGGG**AGTTCGATCGGTAGCGGGAGCG-3′ contained T7 promoter sequence (shown in bold) and 5′-CGGGGTGTGtcGGTAACTAGG -3′) or 3 and 4 (5′-CCTAGTTACCgaCACACCCCG-3′ and 5′-CCAAGCTTATTTAAGACCTTTATTAACAGG-3′). The resulting fragments were combined using the overlapping regions and the flanking primers 1 and 4. The overlapping primers contained a mutation (shown in lower case characters) leading to substitution of GAC for AUC within *YB-1* mRNA.

The plasmids pBluescript II SK_YB-1alter_long and pBluescript II SK-YB-1alter_short were obtained by ligation of pBluescript II SK-YB-1WT treated with XhoI and XagI with PCR products amplified using the total HeLa cDNA as template and primers 5′-TCCCTCACGTGCTCTCCGTC-3′ (for long form) or 5′-TGGAGAGAAAGGGCTGTCAGGTG-3′ (for short form) and 5′-CCAAGCTTATTT AAGACCTTTATTAACAGG-3′ and treated with the same restriction endonucleases.

pBluescript II SK_(AUG → AGA)_YB-1alter was obtained using site-specific mutagenesis by overlap extension. Two PCR products were amplified using the plasmid pBluescript II SK_YB-1alter_long as template and primers 1 and 2 (5′-TCCCTCACGTGCTCTCCGTC-3′ and 5′-GGCTTGGGtcTCCGGTC-3′) or 3 and 4 (5′-GACCGGAgaCCCAAGCC-3′ and 5′-CCCATAGGGTCTCCGCATG-3′). The resulting fragments were combined using the overlapping regions and the flanking primers 1 and 4. The overlapping primers contained a mutation (shown in lower case characters) leading to substitution of AGA for AUG within the alternative form of *YB-1* cDNA. The resulting PCR product was ligated with pJET 1.2 vector. The ≈700 bp DNA fragment obtained by treating this intermediate construct with XhoI and XagI was ligated with the plasmid pBluescript II SK *YB-1* WT treated with the same restriction endonucleases.

pBluescript II SK_(UAG(stop) →CAG)_YB-1alter was obtained using site-specific mutagenesis by overlap extension. Two PCR products were amplified using the plasmid pBluescript II SK_YB-1alter_long as template and primers 1 and 2 (5′- TCCCTCACGTGCTCTCCGTC-3′ and 5′-CGATTACCAGCTgTCCTTTTCCA-3′) or 3 and 4 (5′-TGGAAAAGGAcAGCTGGTAATCG-3′ and 5′-CCCATAGGGTCTCCGCATG-3′). The resulting fragments were combined using the overlapping regions and the flanking primers 1 and 4. The overlapping primers contained a mutation (shown in lower case characters) leading to substitution of CAG for the UAG within the alternative form of *YB-1* cDNA. The resulting PCR product was ligated with pJET 1.2 vector. The ≈700 bp DNA fragment obtained by treating this intermediate construct with XhoI and XagI was ligated with the plasmid pBluescript II SK *YB-1* WT treated with the same restriction endonucleases.

pBluescript II SK_(AUCGUG→AUUUUG)_YB-1alter was obtained using site-specific mutagenesis by overlap extension. Two PCR products were amplified using the plasmid pBluescript II SK_YB-1alter_long as template and primers 1 and 2 (5′-TCCCTCACGTGCTCTCCGTC -3′ and 5′-CAAAACAAGCCAaaATTACCAGC-3′) or 3 and 4 (5′-GCTGGTAATttTGGCTTGTTTTG-3′ and 5′-CCCATAGGGTCTCCGCATG-3′). The resulting fragments were combined using the overlapping regions and the flanking primers 1 and 4. The overlapping primers contained a mutation (shown in lower case characters) leading to substitution of AUUUUG for AUCGUG within the alternative form of *YB-1* cDNA. The resulting PCR product was ligated with pJET 1.2 vector. The ≈700 bp DNA fragment obtained by treating this intermediate construct with XhoI and XagI was ligated with the plasmid pBluescript II SK *YB-1* WT treated with the same restriction endonucleases.

### *In vitro* transcriptio

The transcription was performed as described previously [23]. Truncated (103 nt 5′ UTR) *YB-1* mRNA was transcribed by T7 RNA polymerase from plasmid pBluescript II SK-YB-1 WT, linearized with BamHI, or from the corresponding PCR product with T7 promoter.

Full-length (140 nt 5′ UTR) *YB-1* mRNA, truncated (72 nt 5′ UTR) *YB-1* mRNA, truncated (36 nt 5′ UTR) *YB-1* mRNA, and AUC→GAC –(60–58) *YB-1* mRNA were transcribed by T7 RNA polymerase from the corresponding PCR product with T7 promoter.

Long and short alternative forms of *YB-1* mRNA (alter *YB-1* mRNA) were transcribed by T7 RNA polymerase from plasmids pBluescript II SK-YB-1alter_long and pBluescript II SK-YB-1alter_short, respectively, linearized with BamHI.

Mutated forms of alternative *YB-1* mRNA: (AUG→AGA) *aYB-1* mRNA, (UAG(stop) →CAG) *aYB-1* mRNA and (AUCGUG→AUUUUG) *aYB-1* mRNA were transcribed by T7 RNA polymerase from plasmids pBluescript II SK-(AUG→AGA) YB-1alter, pBluescript II SK-(UAG(stop) →CAG)YB-1alter, and pBluescript II SK-(AUCGUG→AUUUUG)YB-1alter, respectively, linearized with BamHI.

Truncated *YB-1* mRNA 5′ UTR fragment (103 nt) was transcribed by T7 RNA polymerase from plasmid pJET 1.2-5′-UTR YB-1_truncated linearized with NcoI.

Full-length *YB-1* mRNA 5′ UTR fragment (140 nt) was transcribed by T7 RNA polymerase from plasmid pJET 1.2-5′-UTR YB-1_full linearized with NcoI.

*YB-1* mRNA coding region fragment (135 nt) was transcribed by T7 RNA polymerase from plasmid pJET 1.2 YB-1_coding region linearized with EheI.

A 100-nucleotide nonspecific RNA fragment was transcribed by T7 RNA polymerase from pBluescript II SK vector linearized with KpnI.

*AβG*β-*globin* RNA of about 1200 nt in length with actin leader and a *GFP (AβG)* fragment was transcribed by T7 RNA polymerase from pUC18 AβG (kindly provided by I.N. Shatsky) linearized with HindIII.

Capped mRNA transcripts were obtained using the ScriptCap m^7^G Capping System and ScriptCap 2′-O-Methyltransferase Enzyme (CELLSCRIPT) according to the manufacturer's recommendations.

### Isolation of rabbit reticulocyte lysate proteins using biotinylated RNA

Proteins were isolated as described previously [4].

### Western blot analysis and antibodies

Proteins were separated by SDS-PAGE and transferred onto a nitrocellulose membrane. The membrane was blocked for 1 h at room temperature with nonfat 5% milk in TBS and incubated overnight at 4°C in TBS-T supplemented with BSA (5%) and appropriate antibodies (polyclonal rat antibody against 14-aminoacid C-terminal peptide of YB-1 (IMTEK, Russia)). Then the membrane was washed for 5 min three times with TBS-T at room temperature and incubated for 1 h at room temperature in TBS-T supplemented with secondary anti-rat antibodies conjugated with HRP (Sigma).

Immunocomplexes were detected using an ECL Prime kit (GE Healthcare) according to the manufacturer's recommendations.

### *In vitro* translation assays

Translation of exogenous mRNA in a rabbit reticulocyte cell-free system was performed as described elsewhere [24]. The incubation mixture (15 µl) contained reticulocyte lysate (7.5 µl), 10 mM Hepes-KOH, pH 7.6, 100 mM KOAc, 1 mM Mg(OAc)_2_, 8 mM creatine phosphate, 0.5 mM spermidine, 0.2 mM GTP, 0.8 mM ATP, 1 mM dithiothreitol, and 25 µM of each of 20 amino acids except for the labeled one, and 10 µM [^35^S]-Met (37.0 TBq)/mmol (Perkin Elmer) was added. Appropriate mRNA was added to the mixture as indicated in the figure legends. Translation was carried out at 30°C for 1 h. [^35^S]-Met-incorporating proteins were analyzed by 12% SDS-PAGE followed by autoradiography. The [^35^S]-labeled proteins were detected using a Packard Cyclone Storage Phosphor System (Packard Instrument Company, Inc.).

### Analysis of mRNA distribution between polysomes and free mRNPs

Cells were washed twice with ice-cold PBS containing 0.1 mg/ml cycloheximide and lysed directly on the plate after addition of 400 µl of polysome extraction buffer: 15 mM Tris-HCl, pH 7.4, 15 mM MgCl_2_, 0.3 M NaCl, 1% Triton X-100, 0.1 mg/ml cycloheximide,1 mg/ml heparin, and 0.2 mM VRC (vanadyl ribonucleoside complex). Extracts were transferred into 1.5 ml tubes and incubated on ice for 10 min with occasional mixing. The nuclei and debris were removed by centrifugation at 12,000 *g* for 10 min in a microcentrifuge. Supernatants were recovered, and 200 µl aliquots were layered onto 50 µl of 50% sucrose cushion composed of extraction buffer lacking Triton X-100 and pelleted at 90,000 rpm for 13 min in a TLA-100 rotor (Beckman) at 4°C. RNA from supernatant (free mRNPs) and pellet (polysomal mRNPs) were isolated by TRIzol and analyzed by RT-PCR.

### Immunoprecipitation (IP)

For IP, translation reaction mixtures were diluted 100-fold and incubated with appropriate antibodies (polyclonal rat antibody against 14-aminoacid C-terminal peptide of YB-1 (IMTEK, Russia) or rat preimmune antibodies, 100 µg each) immobilized on protein G-Sepharose beads (GE Healthcare) for 2 h at 4°C. After extensive washing with PBS, the proteins were eluted with acid–urea sample buffer (8 M urea, 5% acetic acid, 0.025 methylene blue), and analyzed by acid–urea 10% polyacrylamide gel electrophoresis and autoradiography. The [^35^S]-labeled proteins were detected using a Packard Cyclone Storage Phosphor System (Packard Instrument Company, Inc.).

## Supporting Information

Figure S1 **Western-blot analysis of cell lysates using highly specific antibodies against 14-amino acid C-terminal peptide of YB-1.** 15 µg of total protein from rabbit reticulocyte lysate (lane 1) or MCF7 cell lysate (lane 2) was analyzed.
(PPTX)

Figure S2 **Nucleotide sequence of a fragment of alternative** ***YB-1*** **cDNA from human MCF7 (A) and HEK293 (B) cells.** The sequenced chains are identical.
(PPTX)

Figure S3 **Assembly and toeprinting of 48 S and 80 S translation initiation complexes in nuclease-treated rabbit reticulocyte lysate (RRL) with alternative** ***YB-1*** **mRNA.** The toeprint assay was performed as described previously (Dmitriev et al., 2003. *FEBS letters*, 533, 99–104). To assemble the translation initiation complexes, a nuclease-treated RRL was employed. First, a 10 µl master mix containing 7 µl of RRL, 10 U of ribonuclease inhibitor RiboLock (Fermentas), and 10 mM MgAc_2_ was prepared. To assemble 48 S and 80 S initiation complexes, 0.4 µl of 50 mM guanylimidodiphosphate (GMP-PNP) water solution, 0.4 µl of 50 mM MgAc_2_ and 1.2 µl of water or 1 µl of water solution of cycloheximide (10 mg/ml) and 1 µl of water were added to this master mix. In control experiment, 1 µl of water solution of 20 mM cap analog (^7^mGpppG that inhibits 40 S binding to mRNA) and 0.4 µl of 50 mM MgAc_2_ (2 mM) were added. The mixtures were incubated for 5 min at 30 °C, followed by addition of 0.5 µl of mRNA (1 pmol/µl) annealed with 1 µl of [^32^P]-labeled toeprint primer (5 pmol), 8 µl of RT-Mix (25 mM MgAc_2_, 1.25 mM dNTPs, 25 mM Tris–HCl, pH 7.5, 125 mM KCl, 1.25 mM dithiothreitol), 4.4 µl of water and 0.1 µl of RevertAid Premium reverse transcriptase (Fermentas, 200 U/µl) per each assay, and the samples were incubated for 15 min at 30°C. The mixtures were carefully phenol-purified, cDNA products were precipitated with ethanol overnight and finally analyzed using a 6% sequencing gel. The primer 5′-GGTGTACAAATACATCTTCCTTGGTGTC-3′ complementary to exon 3 of the long alternative *YB-1* mRNA sequence was used. cDNA products were compared with a dideoxynucleotide sequence ladder obtained by using the same primer and the appropriate plasmid DNA.
(PPTX)

Figure S4 **YB-1 transcription initiation region studied by HeliScopeCAGE (Forrest et al., 2014; Nature).** Data for several selected cell types are shown. The minor TSSs in the intronic region show visible activity. The X axis corresponds to the genomic coordinates with the gene structure shown at the top panel. The height of the bars is proportional to the number of mRNAs transcribed from a particular position.
(PPTX)

## References

[1] Eliseeva IA, Kim ER, Guryanov SG, Ovchinnikov LP, Lyabin DN (2011) Y-box-binding protein 1 (YB-1) and its functions. Biochemistry (Mosc) 76: 1402–1433. 22339596 10.1134/S0006297911130049

[2] Lasham A, Print CG, Woolley AG, Dunn SE, Braithwaite AW (2013) YB-1: oncoprotein, prognostic marker and therapeutic target? Biochem J 449: 11–23. 23216250 10.1042/BJ20121323

[3] Eliseeva IA, Ovchinnikov LP, Lyabin DN (2012) Specific PABP effect on translation of YB-1 mRNA is neutralized by polyadenylation through a “mini-loop” at 3′ UTR. RNA Biol 9.. 10.4161/rna.22711 23134843

[4] Lyabin DN, Eliseeva IA, Skabkina OV, Ovchinnikov LP (2011) Interplay between Y-box-binding protein 1 (YB-1) and poly(A) binding protein (PABP) in specific regulation of YB-1 mRNA translation. RNA Biol 8.. 10.4161/rna.8.5.16022 PMC3256357 21788731

[5] Lyabin DN, Nigmatullina LF, Doronin AN, Eliseeva IA, Ovchinnikov LP (2013) Identification of proteins specifically interacting with YB-1 mRNA 3′ UTR and the effect of hnRNP Q on YB-1 mRNA translation. Biochemistry (Mosc) 78: 651–659. 23980891 10.1134/S0006297913060102

[6] Skabkina OV, Lyabin DN, Skabkin MA, Ovchinnikov LP (2005) YB-1 autoregulates translation of its own mRNA at or prior to the step of 40S ribosomal subunit joining. Mol Cell Biol 25: 3317–3323. 15798215 10.1128/MCB.25.8.3317-3323.2005 PMC1069629

[7] Fukuda T, Ashizuka M, Nakamura T, Shibahara K, Maeda K, et al (2004) Characterization of the 5′-untranslated region of YB-1 mRNA and autoregulation of translation by YB-1 protein. Nucleic Acids Res 32: 611–622. 14752049 10.1093/nar/gkh223 PMC373347

[8] Lyabin DN, Eliseeva IA, Ovchinnikov LP (2012) YB-1 synthesis is regulated by mTOR signaling pathway. PLoS One 7: e52527. 23285076 10.1371/journal.pone.0052527 PMC3527543

[9] Hsieh AC, Liu Y, Edlind MP, Ingolia NT, Janes MR, et al (2012) The translational landscape of mTOR signalling steers cancer initiation and metastasis. Nature 485: 55–61. 22367541 10.1038/nature10912 PMC3663483

[10] Harrow J, Frankish A, Gonzalez JM, Tapanari E, Diekhans M, et al (2012) GENCODE: the reference human genome annotation for The ENCODE Project. Genome Res 22: 1760–1774. 22955987 10.1101/gr.135350.111 PMC3431492

[11] Yamashita R, Sugano S, Suzuki Y, Nakai K (2012) DBTSS: DataBase of Transcriptional Start Sites progress report in 2012. Nucleic Acids Res 40: D150–154. 22086958 10.1093/nar/gkr1005 PMC3245115

[12] Forrest AR, Kawaji H, Rehli M, Baillie JK, de Hoon MJ, et al (2014) A promoter-level mammalian expression atlas. Nature 507: 462–470. 24670764 10.1038/nature13182 PMC4529748

[13] Meyer LR, Zweig AS, Hinrichs AS, Karolchik D, Kuhn RM, et al (2013) The UCSC Genome Browser database: extensions and updates 2013. Nucleic Acids Res 41: D64–69. 23155063 10.1093/nar/gks1048 PMC3531082

[14] Eliseeva IA VI, Babeyev KE, Buyanova SM, Sysoeva MA, Kondrashov FA, et al (2013) In silico motif analysis suggests an interplay of transcriptional and translational control in mTOR response. Translation 1: e27469. 26955507 10.4161/trla.27469 PMC4718056

[15] Skabkina OV, Skabkin MA, Popova NV, Lyabin DN, Penalva LO, et al (2003) Poly(A)-binding protein positively affects YB-1 mRNA translation through specific interaction with YB-1 mRNA. J Biol Chem 278: 18191–18198. 12646583 10.1074/jbc.M209073200

[16] Kanamori-Katayama M, Itoh M, Kawaji H, Lassmann T, Katayama S, et al (2011) Unamplified cap analysis of gene expression on a single-molecule sequencer. Genome Res 21: 1150–1159. 21596820 10.1101/gr.115469.110 PMC3129257

[17] Carninci P, Sandelin A, Lenhard B, Katayama S, Shimokawa K, et al (2006) Genome-wide analysis of mammalian promoter architecture and evolution. Nat Genet 38: 626–635. 16645617 10.1038/ng1789

[18] Evdokimova VM, Wei CL, Sitikov AS, Simonenko PN, Lazarev OA, et al (1995) The major protein of messenger ribonucleoprotein particles in somatic cells is a member of the Y-box binding transcription factor family. J Biol Chem 270: 3186–3192. 7852402 10.1074/jbc.270.7.3186

[19] Evdokimova V, Tognon CE, Sorensen PH (2012) On translational regulation and EMT. Semin Cancer Biol 22: 437–445. 22554796 10.1016/j.semcancer.2012.04.007

[20] Ingolia NT, Lareau LF, Weissman JS (2011) Ribosome profiling of mouse embryonic stem cells reveals the complexity and dynamics of mammalian proteomes. Cell 147: 789–802. 22056041 10.1016/j.cell.2011.10.002 PMC3225288

[21] Lee S, Liu B, Huang SX, Shen B, Qian SB (2012) Global mapping of translation initiation sites in mammalian cells at single-nucleotide resolution. Proc Natl Acad Sci U S A 109: E2424–2432. 22927429 10.1073/pnas.1207846109 PMC3443142

[22] David JJ, Subramanian SV, Zhang A, Willis WL, Kelm RJ Jr, et al (2012) Y-box binding protein-1 implicated in translational control of fetal myocardial gene expression after cardiac transplant. Exp Biol Med (Maywood) 237: 593–607. 22619371 10.1258/ebm.2012.011137

[23] Pokrovskaya ID, Gurevich VV (1994) *In vitro* transcription: preparative RNA yields in analytical scale reactions. Anal Biochem 220: 420–423. 7526740 10.1006/abio.1994.1360

[24] Pelham HR, Jackson RJ (1976) An efficient mRNA-dependent translation system from reticulocyte lysates. Eur J Biochem 67: 247–256. 823012 10.1111/j.1432-1033.1976.tb10656.x

