# Supplementary material for: Alternative Forms of Y-Box Binding Protein 1 and YB-1 mRNA
Source: PLoS One. 2014 Aug 12;9(8):e104513. doi: 10.1371/journal.pone.0104513 (PMC4130533; doi:10.1371/journal.pone.0104513)
Supplement: Figure S1 — Western-blot analysis of cell lysates using highly specific antibodies against 14-amino acid C-terminal peptide of YB-1. 15 µg of total protein from rabbit reticulocyte lysate (lane 1) or MCF7 cell lysate (lane 2) was analyzed. (PPTX) [file pone.0104513.s001.pptx]

## Slide 1
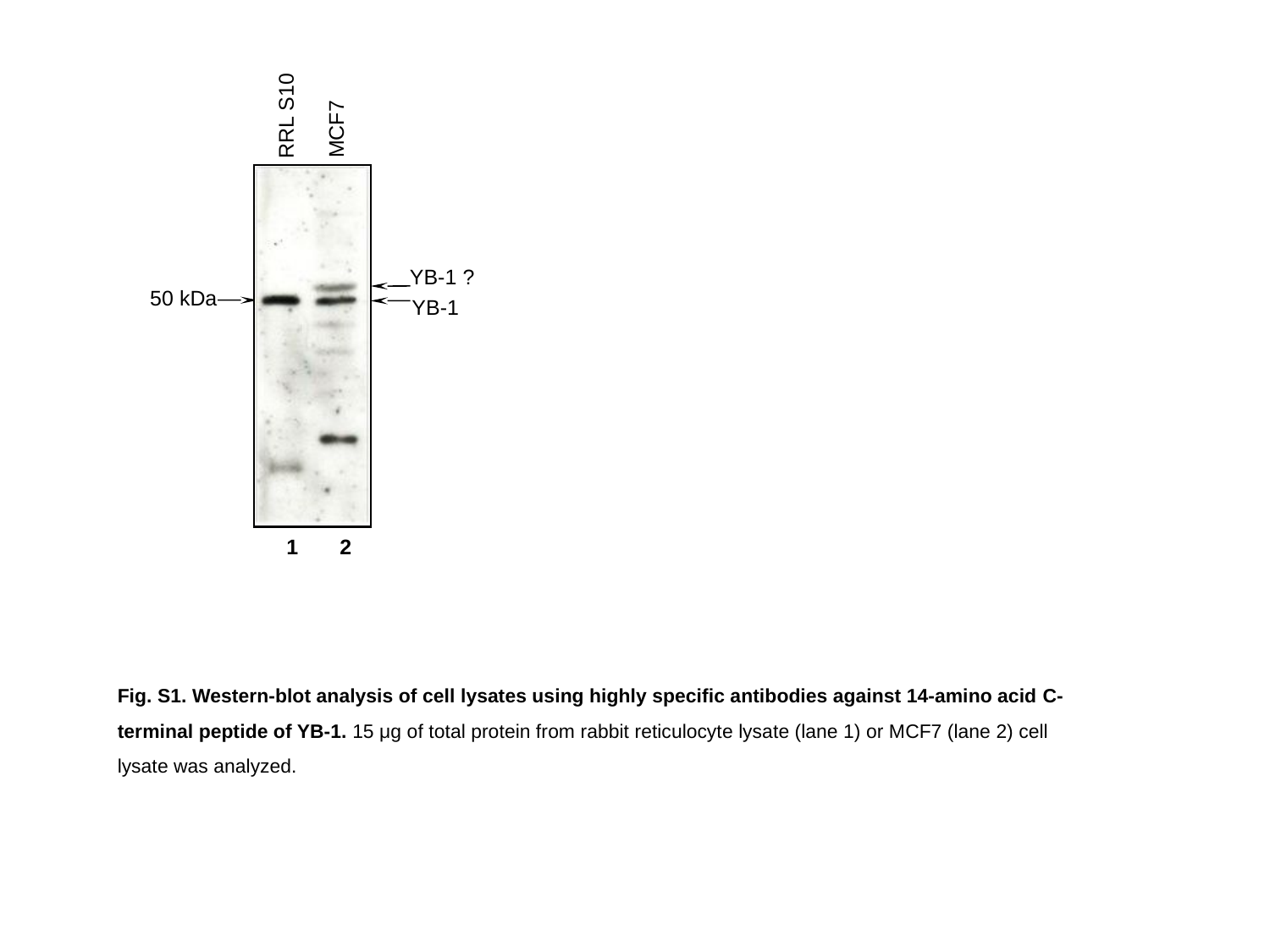

RRL S10
MCF7
YB-1 ?
50 kDa
YB-1
1
2
Fig. S1. Western-blot analysis of cell lysates using highly specific antibodies against 14-amino acid С-terminal peptide of YB-1. 15 μg of total protein from rabbit reticulocyte lysate (lane 1) or MCF7 (lane 2) cell lysate was analyzed.
