# Supplementary material for: Alternative Forms of Y-Box Binding Protein 1 and YB-1 mRNA
Source: PLoS One. 2014 Aug 12;9(8):e104513. doi: 10.1371/journal.pone.0104513 (PMC4130533; doi:10.1371/journal.pone.0104513)
Supplement: Figure S2 — Nucleotide sequence of a fragment of alternative YB-1 cDNA from human MCF7 (A) and HEK293 (B) cells. The sequenced chains are identical. (PPTX) [file pone.0104513.s002.pptx]

## Slide 1
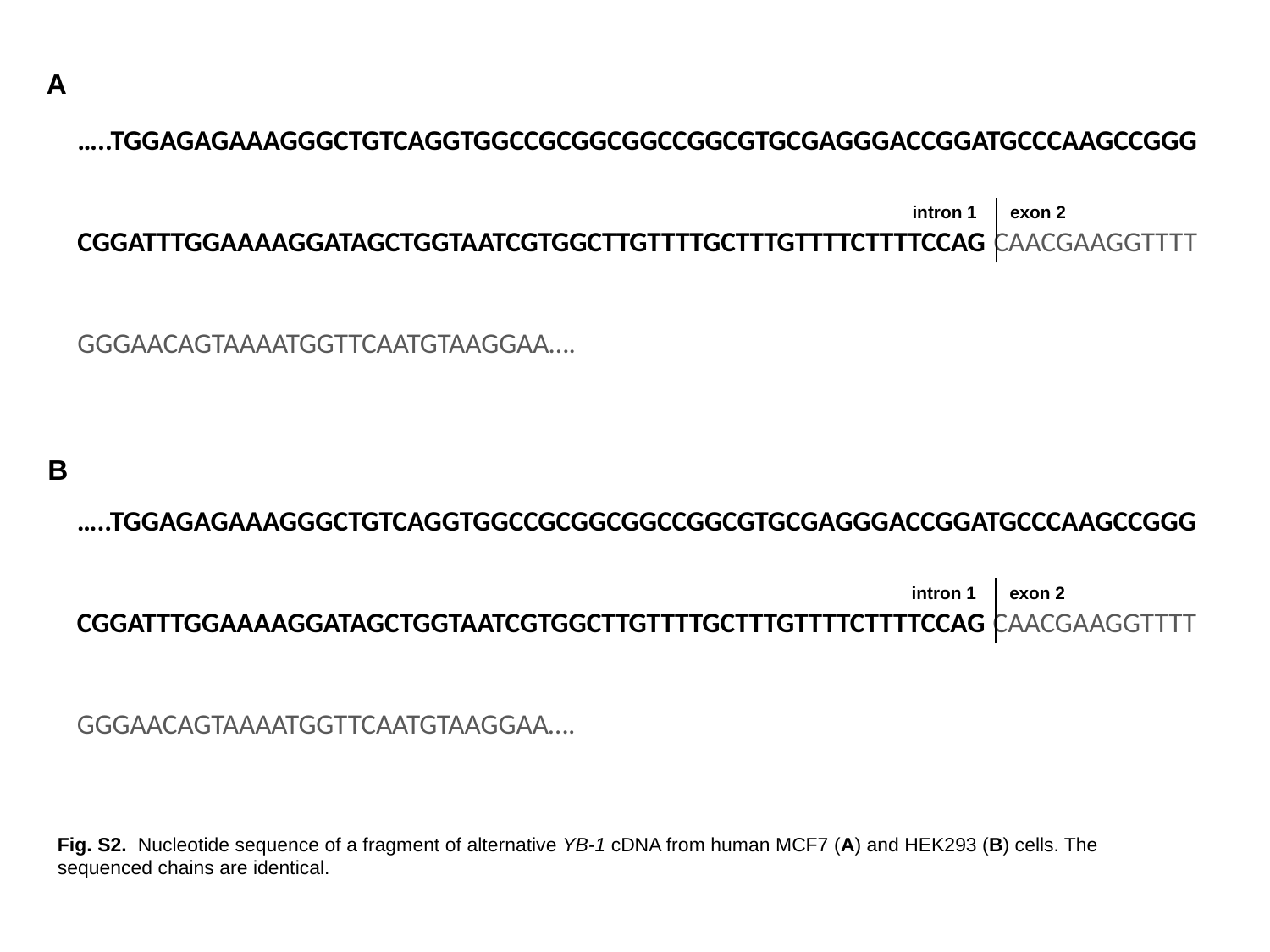

А
…..TGGAGAGAAAGGGCTGTCAGGTGGCCGCGGCGGCCGGCGTGCGAGGGACCGGATGCCCAAGCCGGG
CGGATTTGGAAAAGGATAGCTGGTAATCGTGGCTTGTTTTGCTTTGTTTTCTTTTCCAG CAACGAAGGTTTT
GGGAACAGTAAAATGGTTCAATGTAAGGAA….
intron 1
exon 2
B
…..TGGAGAGAAAGGGCTGTCAGGTGGCCGCGGCGGCCGGCGTGCGAGGGACCGGATGCCCAAGCCGGG
CGGATTTGGAAAAGGATAGCTGGTAATCGTGGCTTGTTTTGCTTTGTTTTCTTTTCCAG CAACGAAGGTTTT
GGGAACAGTAAAATGGTTCAATGTAAGGAA….
intron 1
exon 2
Fig. S2. Nucleotide sequence of a fragment of alternative YB-1 cDNA from human MCF7 (A) and HEK293 (B) cells. The sequenced chains are identical.
