# Supplementary material for: Alternative Forms of Y-Box Binding Protein 1 and YB-1 mRNA
Source: PLoS One. 2014 Aug 12;9(8):e104513. doi: 10.1371/journal.pone.0104513 (PMC4130533; doi:10.1371/journal.pone.0104513)
Supplement: Figure S3 — Assembly and toeprinting of 48 S and 80 S translation initiation complexes in nuclease-treated rabbit reticulocyte lysate (RRL) with alternative YB-1 mRNA. The toeprint assay was performed as described previously (Dmitriev et al., 2003. FEBS letters, 533, 99–104). To assemble the translation initiation complexes, a nuclease-treated RRL was employed. First, a 10 µl master mix containing 7 µl of RRL, 10 U of ribonuclease inhibitor RiboLock (Fermentas), and 10 mM MgAc2 was prepared. To assemble 48 S and 80 S initiation complexes, 0.4 µl of 50 mM guanylimidodiphosphate (GMP-PNP) water solution, 0.4 µl of 50 mM MgAc2 and 1.2 µl of water or 1 µl of water solution of cycloheximide (10 mg/ml) and 1 µl of water were added to this master mix. In control experiment, 1 µl of water solution of 20 mM cap analog (7mGpppG that inhibits 40 S binding to mRNA) and 0.4 µl of 50 mM MgAc2 (2 mM) were added. The mixtures were incubated for 5 min at 30 °C, followed by addition of 0.5 µl of mRNA (1 pmol/µl) annealed with 1 µl of [32P]-labeled toeprint primer (5 pmol), 8 µl of RT-Mix (25 mM MgAc2, 1.25 mM dNTPs, 25 mM Tris–HCl, pH 7.5, 125 mM KCl, 1.25 mM dithiothreitol), 4.4 µl of water and 0.1 µl of RevertAid Premium reverse transcriptase (Fermentas, 200 U/µl) per each assay, and the samples were incubated for 15 min at 30°C. The mixtures were carefully phenol-purified, cDNA products were precipitated with ethanol overnight and finally analyzed using a 6% sequencing gel. The primer 5′-GGTGTACAAATACATCTTCCTTGGTGTC-3′ complementary to exon 3 of the long alternative YB-1 mRNA sequence was used. cDNA products were compared with a dideoxynucleotide sequence ladder obtained by using the same primer and the appropriate plasmid DNA. (PPTX) [file pone.0104513.s003.pptx]

## Slide 1
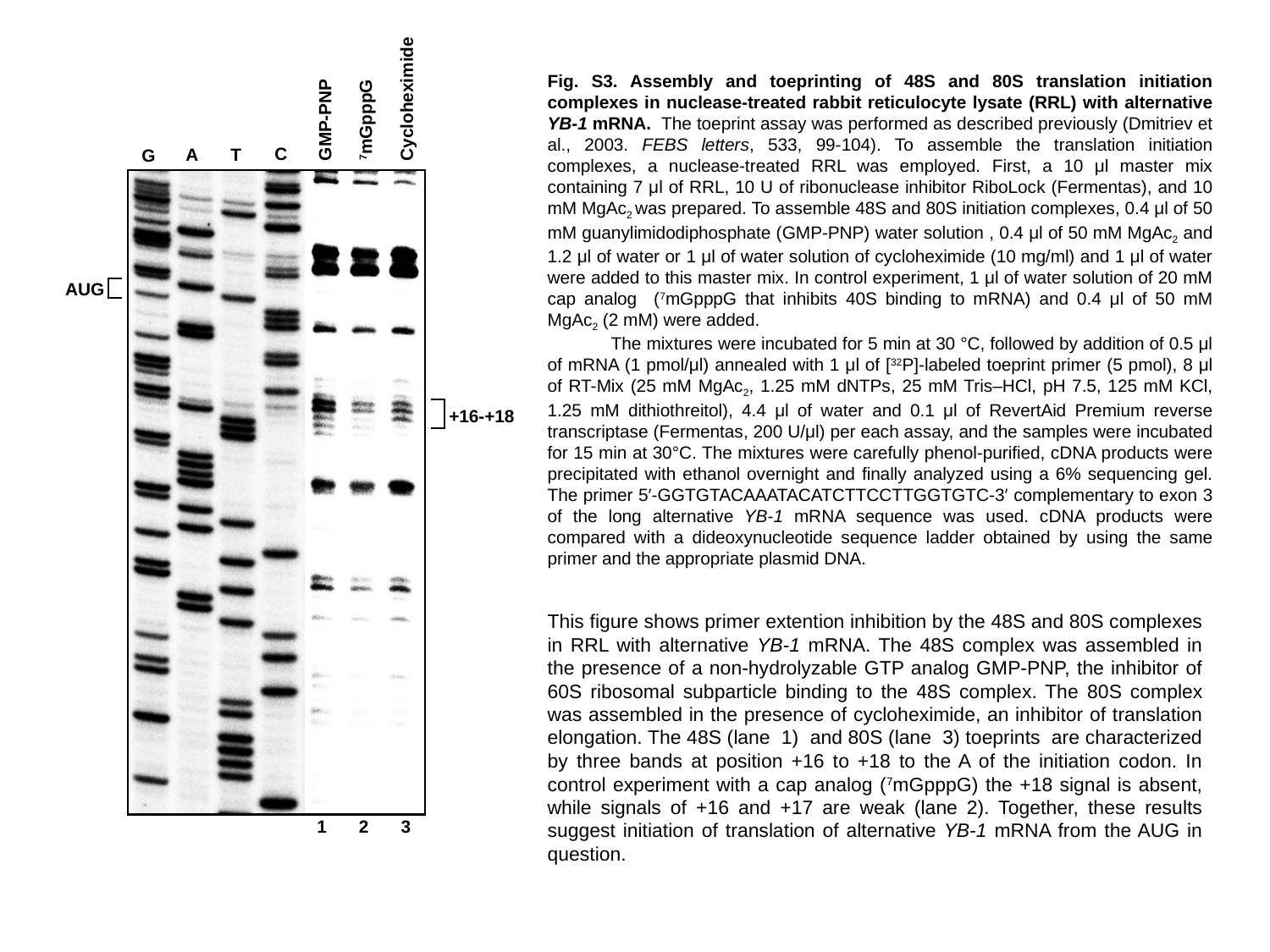

Cycloheximide
7mGpppG
GMP-PNP
C
T
A
G
AUG
+16-+18
Fig. S3. Assembly and toeprinting of 48S and 80S translation initiation complexes in nuclease-treated rabbit reticulocyte lysate (RRL) with alternative YB-1 mRNA. The toeprint assay was performed as described previously (Dmitriev et al., 2003. FEBS letters, 533, 99-104). To assemble the translation initiation complexes, a nuclease-treated RRL was employed. First, a 10 μl master mix containing 7 μl of RRL, 10 U of ribonuclease inhibitor RiboLock (Fermentas), and 10 mM MgAc2 was prepared. To assemble 48S and 80S initiation complexes, 0.4 μl of 50 mM guanylimidodiphosphate (GMP-PNP) water solution , 0.4 μl of 50 mM MgAc2 and 1.2 μl of water or 1 μl of water solution of cycloheximide (10 mg/ml) and 1 μl of water were added to this master mix. In control experiment, 1 μl of water solution of 20 mM cap analog (7mGpppG that inhibits 40S binding to mRNA) and 0.4 μl of 50 mM MgAc2 (2 mM) were added.
The mixtures were incubated for 5 min at 30 °C, followed by addition of 0.5 μl of mRNA (1 pmol/μl) annealed with 1 μl of [32P]-labeled toeprint primer (5 pmol), 8 μl of RT-Mix (25 mM MgAc2, 1.25 mM dNTPs, 25 mM Tris–HCl, pH 7.5, 125 mM KCl, 1.25 mM dithiothreitol), 4.4 μl of water and 0.1 μl of RevertAid Premium reverse transcriptase (Fermentas, 200 U/μl) per each assay, and the samples were incubated for 15 min at 30°C. The mixtures were carefully phenol-purified, cDNA products were precipitated with ethanol overnight and finally analyzed using a 6% sequencing gel. The primer 5′-GGTGTACAAATACATCTTCCTTGGTGTC-3′ complementary to exon 3 of the long alternative YB-1 mRNA sequence was used. cDNA products were compared with a dideoxynucleotide sequence ladder obtained by using the same primer and the appropriate plasmid DNA.
This figure shows primer extention inhibition by the 48S and 80S complexes in RRL with alternative YB-1 mRNA. The 48S complex was assembled in the presence of a non-hydrolyzable GTP analog GMP-PNP, the inhibitor of 60S ribosomal subparticle binding to the 48S complex. The 80S complex was assembled in the presence of cycloheximide, an inhibitor of translation elongation. The 48S (lane 1) and 80S (lane 3) toeprints are characterized by three bands at position +16 to +18 to the A of the initiation codon. In control experiment with a cap analog (7mGpppG) the +18 signal is absent, while signals of +16 and +17 are weak (lane 2). Together, these results suggest initiation of translation of alternative YB-1 mRNA from the AUG in question.
1
2
3
