# Supplementary material for: Alternative Forms of Y-Box Binding Protein 1 and YB-1 mRNA
Source: PLoS One. 2014 Aug 12;9(8):e104513. doi: 10.1371/journal.pone.0104513 (PMC4130533; doi:10.1371/journal.pone.0104513)
Supplement: Figure S4 — YB-1 transcription initiation region studied by HeliScopeCAGE (Forrest et al., 2014; Nature). Data for several selected cell types are shown. The minor TSSs in the intronic region show visible activity. The X axis corresponds to the genomic coordinates with the gene structure shown at the top panel. The height of the bars is proportional to the number of mRNAs transcribed from a particular position. (PPTX) [file pone.0104513.s004.pptx]

## Slide 1
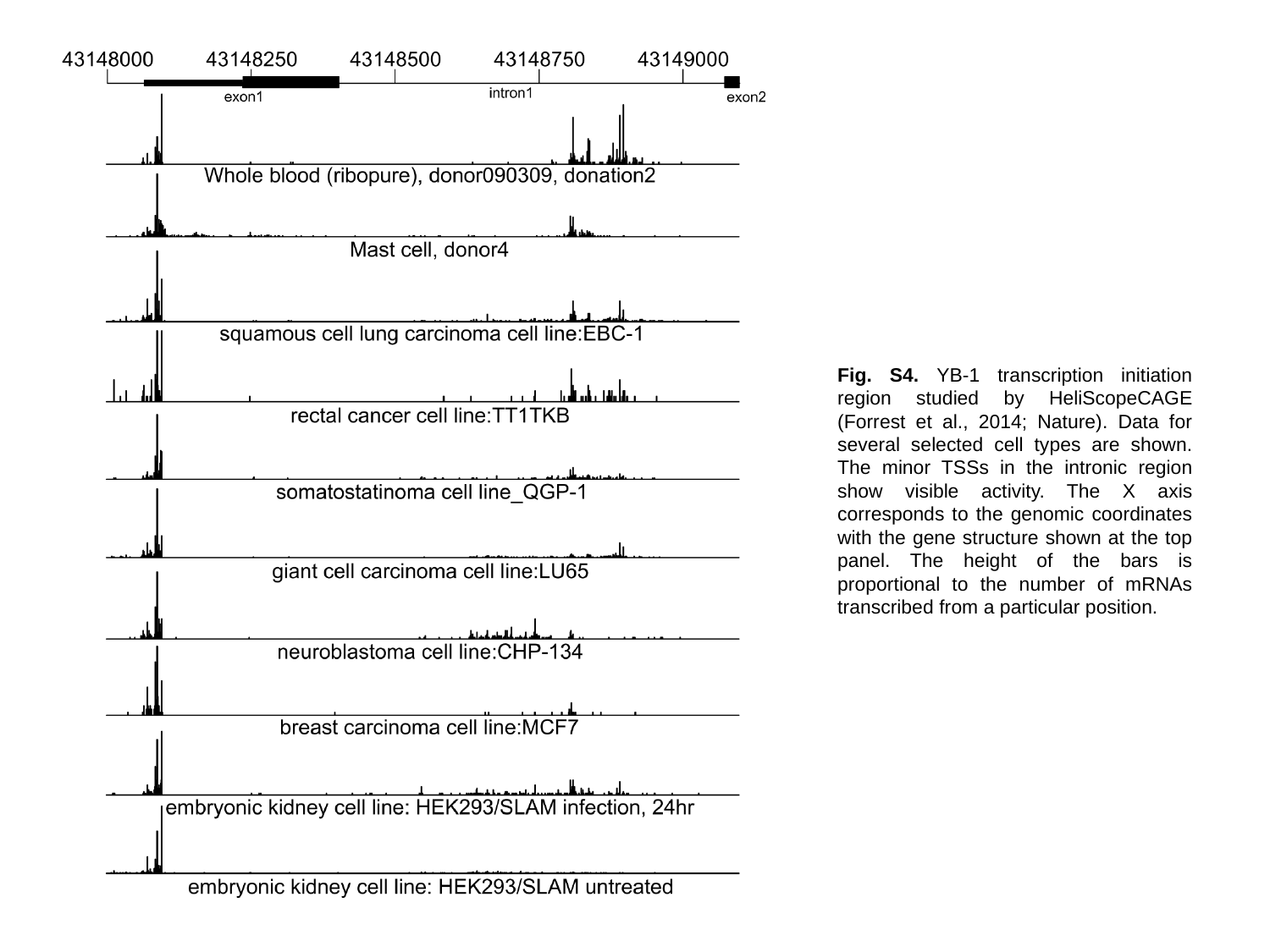

Fig. S4. YB-1 transcription initiation region studied by HeliScopeCAGE (Forrest et al., 2014; Nature). Data for several selected cell types are shown. The minor TSSs in the intronic region show visible activity. The X axis corresponds to the genomic coordinates with the gene structure shown at the top panel. The height of the bars is proportional to the number of mRNAs transcribed from a particular position.
